# Private health insurance policies in Israel: a report on the 2012 Dead Sea Conference

**DOI:** 10.1186/2045-4015-2-25

**Published:** 2013-06-27

**Authors:** Gabi Bin Nun

**Affiliations:** 1 Ben-Gurion University of the Negev, Beer-Sheva, Israel

## Abstract

The private health insurance (commercial and supplementary health insurance) sector has undergone a revolutionary transformation in recent years, both in the number of individuals who own private plans, and in the financial scope of these plans. With these developments in the background, leaders of the Israeli healthcare system convened in December 2012 at the Dead Sea for a discussion on “Private healthcare insurance plans in Israel: Developments, concerns, and directions for a solution”. This meeting report summarizes the main issues discussed at the conference.

## Background

The National Healthcare Insurance Law that was passed in 1995 was designed to guarantee a broad publicly-financed health benefit basket, with services delivered at a high standard, for the entire Israeli population. In addition to this health benefit basket, the Law permitted the sale of supplementary insurance plans, and in 1996, a resolution determined that these plans would be operated directly by the Kupot Holim (the “Sick Funds”) rather than by commercial insurance companies.

Supplementary insurance plans initially included a limited service package (beyond the basic health benefit basket) and its subscribers encompassed 45% of the general population (based on the 1998 summative reports of the Kupat Holim supplemental services). At the time, less than one quarter of the general population was covered in a commercial health insurance plan [1].

The state of the private (supplementary and commercial) health insurance sector at the end of 2012 reflects the revolution that affected the industry, both in terms of the number of individuals covered by private insurance plans, and in terms of the financial scope of these plans. In this period (1998–2011), Israel became one of the leading countries in private healthcare plans—taking fourth place after the USA, France, and Canada [2,3]. In view of these developments, leaders of the Israeli healthcare system convened in December 2012 at the Dead Sea for a discussion on “Private healthcare insurance in Israel: Developments, inter-relationships, concerns, and directions for a solution”.

**Figure 1:**
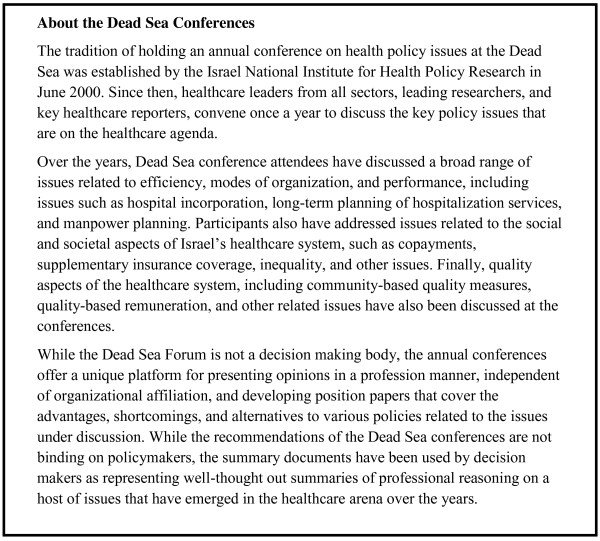
About the Dead Sea Conferences.

This meeting report begins by reviewing the main developments in the private health insurance sector, including the number of subscribers and the financial scope of the insurance plans. The second section of the report offers a brief review of the discussions in the Thirteenth Dead Sea Conference and the main recommendations of its work groups. The complete transcripts of the work group discussions are available in Hebrew in the Proceedings of the Thirteenth Dead Sea Conference, “Private Healthcare Insurance in Israel: Developments, Interactions, Concerns, and Suggestions for a Solution”, published in April 2013 by the Israel National Institute for Health Policy Research [4]. Background information on the Dead Sea Conferences appears in the accompanying Figure 1.

### Developments in the private health insurance sector over the past decade

**Figure 2:**
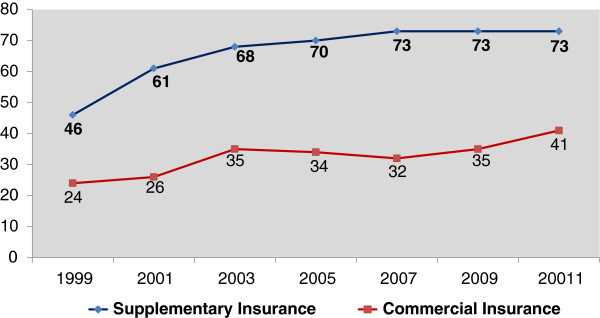
**Individuals covered by supplementary and commercial health insurance (% of total population).** Sources: [5,6].

**Figure 3:**
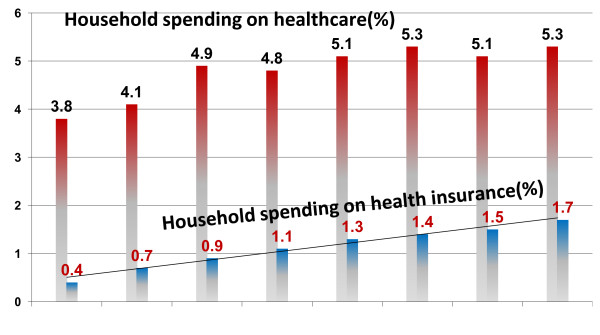
**Household spending on healthcare and health insurance as a % of total household spending.** Source: [7].

The scope of private health insurance in Israel can be measured on two levels: the number of insured individuals (Figure 2) and the financial scope of the insurance plans (Figure 3).

The number of insured individuals in supplementary insurance plans increased from 60% of the total population in 2000 to 74% in 2011 [1,5]. The share of the population covered by commercial insurance plans increased from 25% in 2000 to 41% in 2011 [6].

Notably, another recent trend, in addition to the growing number of subscribers, is the growth in the number of insured individuals’ who have “traded-up” for more extensive insurance coverage

• Private healthcare insurance revenues: Total revenues of the private health insurance plans has also increased. Revenues from supplemental insurance premiums increased fourfold in the span of a decade, from NIS 700 million in 2000 to NIS 3.1 billion in 2011 [5]. In commercial insurance plans, revenues increased 2.5 times in eight years—from NIS 1.1 billion in 2003 to NIS 2.7 billion in 2011 [6].

Another expression of the growing breadth and depth of private health insurance is also reflected in household spending data collected by the Central Bureau of Statistics: In 2000, 18% of total household healthcare spending was designated for private health insurance, while in 2011, household spending on private insurance accounted for 33% of total household healthcare expenses [7].

The conclusion that emerges from these findings is that the private (supplemental and commercial) health insurance sector underwent a revolutionary transformation over the past decade:

• Private (supplemental and commercial) health insurance plans are owned by a growing number of individuals, who tend to purchase increasingly broad coverage over time.

• Insurance premiums and revenues of private health insurance plans are growing.

Thus, Israel now has one of the highest private health insurance ownership rates in the world. Private health insurance plans are no longer a marginal phenomenon: They now account for one-third of total household healthcare spending [7].

One view considers the accelerated growth in private health insurance as an expression of consumers’ autonomy and the rational preferences of risk-averse consumers who assume responsibility for their own health. This trend may also be traced to the growing awareness of health insurance in general, (following the enactment of the Public Health Insurance Law); developments in the collective insurance sector, which operates through employers; and a general increase in the standard of living.

Another view attributes this development to the diminishing financing and the content of the public health benefit basket and the continuous decline in the availability and quality of public services, which have eroded trust in the public healthcare system and promoted the search for alternative private insurance coverage options. The real explanation for this trend is probably a combination of these factors, in addition to aggressive marketing campaigns by health insurance companies, and a misguided belief that an individual’s ability to fully access services in the basic (public) health benefit basket is dependent on whether the individual possesses supplemental or commercial insurance.

### Highlights of the discussions, conclusions, and recommendations of the thirteenth Dead Sea Conference and its three working groups:^1^

Following the format of the previous Dead Sea Conferences, attendees of the Thirteenth Dead Sea Conference participated in three working groups. Highlights of the discussions, recommendations, and conclusions of these groups are presented below.

### Group #1 – The impact of developments in the insurance sector on service delivery

This group discussed the impact of insurance on overuse of medical tests and procedures, physicians’ practice and work hours, and wage differentials among physicians.

Group members generally agreed on the key concerns of the healthcare system but were divided on the underlying causes: Advocates of the public healthcare system attributed most of the problems to the emergence and growth of the private system, which created unfair competition between public and private health service providers. Private healthcare supporters argued, in contrast, that the existence of private healthcare services improved the situation of most of the country’s citizens, by providing an alternative of adequate quality, and that the problems of the public healthcare system should be attributed primarily to reduced public spending on healthcare.

The group’s main conclusions and recommendations included:

• Group members agreed that the growth in private insurance plans occurred against the backdrop of the declining (financing and service levels) of the public healthcare system and the group’s primary recommendation was to strengthen the public system.

• Group members agreed that the public health benefit basket should be enhanced gradually by diverting services from supplemental healthcare plans to the pubic basket, starting with those services whose benefits are greatest relative to their costs.

• Although private health insurance is a voluntary scheme, the group stated that supplemental insurance plans administered by the Kupot Holim (effectively have a public character and therefore these insurance schemes should have more egalitarian features,

• The majority of group members believed that it is important to maintain a separation between the public and private systems (including SHARAP), both in terms of funding and in terms of service delivery, and that this will promote equality and control of national healthcare spending.

### Group #2: State involvement in the regulation of private insurance markets

This group discussed three topics: (a) transparency issues and due disclosure required by the regulator for decision making; (b) the functions of regulation of prices, products, and coverage; (c) boundaries and coordination between the two regulating authorities, the MoH and the MoF Commissioner of Insurance.

Opinions in the group were divided primarily on the appropriate interface between the two regulatory authorities, the MoH and the MoF. According to one view, the current lack of coordination between the two regulatory authorities creates a regulatory vacuum, and responsibility should therefore be shared. Advocates of the opposing position argued that shared responsibility effectively means no responsibility and therefore the division of authority between the two regulators should be more clearly defined.

The group’s main conclusions and recommendations included the following:

• The group recommended creating a comparative data bank on private insurance (on topics such as specific uses, distribution of policies, etc.) to support policy planning and decision making.

• In the area of price regulation, the group recommended examining various options for regulating the prices of private medical service inputs to prevent insurers from attracting clients using below-cost pricing and subsequently increasing premiums. The group also recommended intervening in price determination in cases of uncompetitive markets. In the field of product regulation, the group suggested establishing a mechanism for pre-approving products in voluntary insurance plans and for ensuring that the regulation does not have an adverse impact on innovations or competition.

• In the field of the division of authority between healthcare regulators in the MoF and in the MoH, the group recommended increasing cooperation between both regulators and having them communicate regularly (in the form of consultations, transfer of information, communications directly through the ministries rather than through the media); Group members did not, however, agree where these boundaries should be drawn.

### Group #3: The effects of developments in the private (supplemental and commercial) health insurance sector on consumers

This group discussed three effects of private insurance on consumers: (a) information and due disclosure: potential barriers that prevent consumers from exercising their rights; (b) duplicate insurance: potential situations of duplicate insurance and how to reduce them; (c) the return on consumers’ investments: how to ensure an adequate return on consumers’ investments in the private health insurance market. Differences of opinion were voiced on all three issues, reflecting two basic positions on private health insurance: On the one hand, a position that views health insurance as one more area of insurance that should be subject to regulation, and especially the financial aspects of its operations (actuarial issues, financial stability), and on the other hand, the position that views health insurance as a special field whose societal implications require separate regulation.

The group’s main conclusions and recommendations included:

• Information, due disclosure, and exercise of rights: The group recommended defining guidelines for due disclosure and effective information sharing with consumers (before and after the purchase of insurance), adjusting disclosure to the specific features and needs of various groups of healthcare service consumers, and retaining the option to equalize alternative insurance arrangements, with emphasis on disclosing the intersections between the various insurance layers, and providing information on how consumers may exercise their rights in an insurance event.

• Duplicate insurance – To reduce the practice of duplicate insurance, the group recommended increasing consumer awareness of potential duplicate insurance prior to purchase, through (a) a media campaign on the rights available in the basic health benefit basket, (b) oversight of private insurance marketing practices, (c) prohibition on marketing insurance products that create duplicate coverage, (d) restrictions on the composition/content/terms of insurance policies, and other means.

• Return on investment - In the matter of maximizing return on the consumer’s investment in health insurance, the group recommended revising the measures used to assess consumers’ ROI in health insurance, by developing additional indices that reflect consumer satisfaction (at purchase and at exercise of rights) and service quality (the clinical and the insurance aspects of service quality). To help consumers ensure that their claims are not rejected, the group suggested that the Commissioner of Insurance publish information on rejected claims as well as court decisions that overturned insurance companies’ rejections.

### Summary

Taken together, the recommendations of the three working groups indicates that the groups agreed that the declining public system played a significant role that contributed to the growth of private health insurance, and that the public health benefit basket should be strengthened. Group members also agreed that collaboration between the two regulators in charge of private insurance should be strengthened.

### Endnotes

^1^A broad range of opinions and views were expressed in all three working groups. This brief summary focuses on the majority positions. For a detailed account of the full range of opinions and recommendations, refer to the publication “Private Healthcare Insurance in Israel: Developments, Interactions, Concerns, and Suggestions for a Solution”, published in April 2013 by the Israel National Institute for Health Policy Research [4].

## Competing interests

The author declares that he has no competing interests.
